# Subclinical Hypothyroidism in Danish Lean and Obese Children and Adolescents

**DOI:** 10.4274/jcrpe.3319

**Published:** 2017-03-01

**Authors:** Maria Dahl, Johanne Dam Ohrt, Cilius Esmann Fonvig, Julie Tonsgaard Kloppenborg, Oluf Pedersen, Torben Hansen, Jens-Christian Holm

**Affiliations:** 1 Copenhagen University Hospital Holbæk, The Children’s Obesity Clinic, Department of Pediatrics, Holbæk, Denmark; 2 University of Copenhagen Faculty of Health and Medical Science, The Novo Nordisk Foundation Center for Basic Metabolic Research, Section of Metabolic Genetics, Copenhagen, Denmark; 3 University of Copenhagen, Herlev Hospital, Department of Pediatrics, Herlev, Denmark; 4 University of Southern Denmark, The Faculty of Health Sciences, Odense, Denmark

**Keywords:** Childhood obesity, thyroid hormones, waist-height ratio

## Abstract

**Objective:**

Thyroid abnormalities are common in obese children. The aim of the present study was to examine the prevalence of subclinical hypothyroidism (SH) and to determine how circulating thyroid hormone concentrations correlate with anthropometrics in Danish lean and obese children and adolescents.

**Methods:**

In this cross-sectional study, we included 3006 children and adolescents, aged 6-18 years, from the Registry of the Danish Childhood Obesity Biobank. The overweight/obese group (n=1796) consisted of study participants with a body mass index (BMI) standard deviation score (SDS) ≥1.28. The control group (n=1210) comprised lean children with a BMI SDS <1.28. All participants were characterized by anthropometrics (weight, height, and waist circumference) and fasting serum concentrations of thyroid-stimulating hormone (TSH), free triiodothyronine, and free thyroxine (fT_4_) at baseline.

**Results:**

The prevalence of SH was higher among overweight/obese compared to lean study participants (10.4% vs. 6.4%, p=0.0001). In the overweight/obese group, fasting serum TSH concentrations were associated positively with BMI SDS (p<0.0001) and waist-height ratio (WHtR) (p<0.0001) independent of age, sex, and pubertal developmental stage, whereas fasting serum fT_4_ concentrations were associated positively only with WHtR. The odds ratio of exhibiting SH was 1.8 when being overweight/obese compared with lean (p=0.0007) and 1.8 when presenting with a WHtR >0.5 (p=0.0003).

**Conclusion:**

The prevalence of SH was higher among overweight/obese study participants. The positive correlations of circulating TSH and fT_4_ with WHtR suggest that central obesity, independent of the overall degree of obesity, augments the risk of concurrent thyroid abnormalities in children and adolescents with obesity.

**WHAT IS ALREADY KNOWN ON THIS TOPIC?**

Subclinical hypothyroidism (SH) is a frequent finding in children and adolescents with obesity. Furthermore, SH has been associated with increased risk of exhibiting cardio-metabolic disorders. The mechanisms responsible for the disturbances in thyroid hormone concentrations during obesity are still unclear.

**WHAT THIS STUDY ADDS?**

This study underlines that SH is more prevalent among overweight/obese children compared to their lean peers. We found a positive correlation between waist-height ratio (used as a proxy of central fat accumulation) and fasting serum concentrations of thyroid-stimulating hormone, indicating that central obesity is of importance when evaluating the risk of children with obesity to exhibit thyroid abnormalities.

## INTRODUCTION

Thyroid hormones are known to play an important role in the regulation of thermogenesis (1,2) and are also involved in glucose and lipid metabolism (2,3), making them essential determinants of energy expenditure.

It is well known that overt hypothyroidism can lead to obesity. Nevertheless, modest alterations in thyroid function are more common among obese compared to lean children, including elevated serum concentrations of thyroid-stimulating hormone (TSH) combined with normal serum concentrations of free thyroxine (fT_4_)-often termed subclinical hypothyroidism (SH) (4,5,6,7,8,9). SH is a diagnosis of exclusion which does not cause any clinical symptoms of hypothyroidism. Whether SH in obesity is caused by a reduced thyroid function or reflects an adaptive mechanism against obesity in order to increase energy expenditure remains to be clarified. Longitudinal studies in obese children have reported improvements in thyroid status by reductions in TSH concentrations during weight loss (10,11) supporting the latter notion; however, results have been conflicting (6,12).

SH has been suggested a risk factor for cardiovascular and metabolic disorders such as hypertension and dyslipidemia (3,13,14). Likewise, central fat accumulation in obese children is associated with a range of metabolic comorbidities and development of cardiovascular complications (15). Although there exist various studies evaluating thyroid status in children with obesity, most studies have focused on body mass index (BMI) standard deviation score (SDS) as the anthropometric measure to describe the degree of obesity (5,6,7,14). Few studies have investigated associations with other anthropometric measures such as waist circumference (WC) (16,17) and waist-hip ratio (17). Waist-height ratio (WHtR) has been advocated as an effective and convenient measure of central adiposity in children that could potentially be superior to BMI SDS in estimating cardiovascular and metabolic risk (15,18,19).

The aim of the present study was to determine the prevalence of SH and investigate the fasting serum concentrations of thyroid hormones in children and adolescents with overweight/obesity and in their lean peers. Furthermore, we evaluated how anthropometric measures, including WHtR as a proxy of central adiposity, were associated to fasting serum concentrations of thyroid hormones, as we hypothesized that SH in children could be associated to the degree of central adiposity.

## METHODS

### Study Sample and Design

For this cross-sectional study, we recruited children and adolescents from the Registry of the Danish Childhood Obesity Biobank. From August 2007 to May 2014, 1854 overweight/obese children and adolescents were enrolled in the chronic care multidisciplinary treatment program at The Children’s Obesity Clinic, Department of Pediatrics, Copenhagen University Hospital Holbæk, Denmark. The chronic care multidisciplinary treatment program is furthermore established in eight municipal clinics throughout Denmark wherefrom 946 overweight/obese children and adolescents were registered from June 2012 to May 2014. From June 2011 to May 2014, a control group was recruited comprising a population sample of 1979 Danish school children from schools in the same geographical regions (Capital Region and Region Zealand, Denmark). None of the recruitment areas in Denmark are known to suffer from iodine deficiency. Inclusion criteria for both groups were: i) Age 6-18 years and ii) a fasting blood sample drawn at the time of inclusion into the Biobank or at initiation of obesity treatment. Overall, the overweight/obese group was defined by a BMI SDS ≥1.28, corresponding to the 90^th^ percentile according to a Danish age- and sex-adjusted reference (20,21). For sub-group analyses, overweight was defined as BMI SDS ≥1.28, while obesity was defined as BMI SDS ≥2.33 (20,21). The lean control group consisted of children and adolescents with a BMI SDS <1.28. The exclusion criteria were: i) An intake of medications known to affect serum concentrations of thyroid hormones (22), ii) a time period of more than 60 days between the anthropometric measures and the fasting blood sampling, and iii) a serum TSH concentration above 10.0 mIU/L or below 0.45 mIU/L, both suggestive cut-offs of overt thyroid disease (23,24).

### Methods

Study participants were assessed with concomitant clinical and biochemical characteristics. The children and adolescents recruited from The Children’s Obesity Clinic were assessed at treatment enrollment. Clinical characteristics included age, sex, height, weight, BMI SDS, WC, and pubertal developmental stage. Biochemical characteristics included serum concentrations of TSH, fT_4_, and free triiodothyronine (fT_3_).

### Anthropometry

Anthropometric measures were obtained with the study participants wearing light indoor clothing with empty pockets and without shoes. Height was measured to the nearest mm using a stadiometer. Weight was measured to the nearest 100 grams using a Tanita Digital Medical Scale (WB-100 MA, Tanita Corp., Tokyo, Japan). BMI was calculated as the weight in kilograms divided by the square of the height in meters (kg/m^2^). BMI SDS was calculated using the LMS method (25) by transforming BMI into a normal distribution based on a Danish population with the same sex and age (20). WC was measured to the nearest mm at the end of a normal expiration with a non-elastic measuring tape placed at the level of the umbilicus while the participant was standing with arms down. WHtR was calculated as WC divided by height.

### Pubertal Developmental Stage

Determination of pubertal developmental stage was rated according to the classification of Tanner (26,27). A trained pediatrician examined the children and adolescents with overweight/obesity, while pubertal staging of the lean control group was obtained via a questionnaire with picture pattern recognition of the five different Tanner stages accompanied by a text describing each category.

### Biochemical Measurements

Venous blood samples were drawn from an antecubital vein after an overnight fast. If required, a local anesthetic cream (lidocaine/prilocaine mixture, Emla, AstraZeneca, Stockholm, Sweden) was applied one hour prior to venipuncture. The blood samples were analyzed immediately after venipuncture. A Cobas 6000^®^ analyzer (F. Hoffmann-La Roche Ltd, Basel, Switzerland) was used to analyze serum concentrations of TSH and fT_4_ from August 2007 to May 2013. A Dimension Vista/Centaur analyzer (Siemens, Munich, Germany) was used to measure serum concentrations of TSH, fT_4_, and fT_3_ on blood samples collected after May 2013, making measures of fT_3_ concentrations available in a sub-group of study participants. Both analyzers used immunologic chemiluminescent assays. The analyzers yielded concordant results and were evaluated to be comparable in the analyses of TSH and fT_4_. This was based on a linear relationship (r^2^=0.985 for fT_4_ and r^2^=0.997 for TSH) between the analyzers and a bias of 6% for TSH and 13.7% for fT_4_. Intra-assay coefficients of variation were <0.10 for both analyzers.

Intervals for normal values were set as follows: TSH 0.45-4.5 mIU/L (23), fT_4_ 11.1-23.4 pmol/L, and fT_3_ 3.62-8.71 pmol/L based on analyses of a German pediatric population comprising 722 children and adolescents (28).

SH was defined as an elevated serum concentration of TSH in the range 4.5-10.0 mIU/L combined with a normal serum concentration of fT_4_ (24). Euthyroidism was defined as normal serum concentrations of TSH and fT_4_ alongside no clinical signs of hypothyroidism.

### Statistical Analysis

Data were analyzed as continuous variables between groups by Wilcoxon rank-sum test, and two by two comparisons were analyzed by Fisher’s exact test. Multiple linear regression models where used to investigate associations, and logistic regression models of the binomial family were used in the assessment of odds ratios (ORs). Levels of significance were set at p<0.05. All statistical analyses were performed using “R” statistical software version 3.1.2 (http://www.r-project.org).

### Ethical Aspects

Informed written consent was obtained from study participants aged 18 years and from the parents if the participants were younger. Informed assent was obtained from all children and adolescents. The study received ethical approval by the Ethics Committee of Region Zealand, Denmark (ID no. SJ-104) and by the Danish Data Protection Agency and was performed in accordance with the ethical standards of the Declaration of Helsinki 2013. The present study has been reported in line with the STrengthening the Reporting of OBservational studies in Epidemiology (STROBE) guidelines.

## RESULTS

In total, 3114 recruited children and adolescents fulfilled the inclusion criteria. Of these, 59 subjects were excluded due to intake of medications potentially affecting the thyroid hormones, 47 were excluded due to a period of more than 60 days between anthropometric measures and blood sampling, and two volunteers were excluded due to a serum TSH concentration above 10 mIU/L. The remaining 3006 eligible study participants consisted of 1796 (985 girls) overweight/obese and 1210 (729 girls) lean children and adolescents. Serum fT_3_ concentrations were available in 440 overweight/obese (237 girls) and 534 lean children and youths (318 girls). Overall, the group of overweight/obese children and adolescents had a median BMI SDS of 2.75 [interquartile range (iqr) 2.22-3.22] and a median age of 11.6 (iqr 9.5-13.9), while the median BMI SDS in the lean group was 0.06 (iqr -0.53-0.61) combined with a median age of 12.1 (iqr 9.6-14.9), with the group of lean girls showing a higher age than the other groups. Sex-stratified clinical and biochemical baseline characteristics are shown in Table 1.

The prevalence of SH was higher among overweight/obese compared to lean children and adolescents (10.4% vs. 6.4%, p=0.0001). Stratifying for sex, the SH prevalence was higher in overweight/obese boys than in lean boys, whereas no significant difference in the prevalence rates was observed when comparing overweight/obese and lean girls (Table 1).

**Figure 1:**
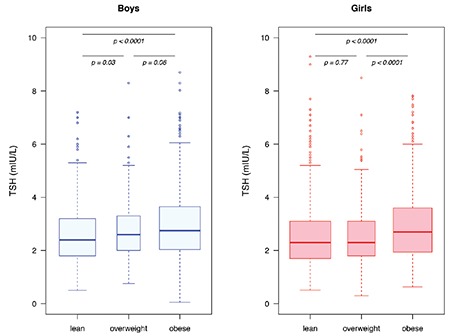
Box-plots showing serum concentrations of thyroid-stimulating hormone in the 3006 Danish lean, overweight, and obese boys and girls. TSH: thyroid-stimulating hormone

Overall, the overweight/obese children and youths exhibited higher concentrations of serum TSH (p<0.0001) and serum fT_4_ (p<0.0001) when compared with lean children and adolescents. Overweight/obese girls had higher serum concentrations of TSH, fT_4_, and fT_3_ compared to lean girls (Table 1). Furthermore, serum TSH concentrations were higher in obese as compared with overweight girls (Figure 1). Serum concentrations of TSH in overweight/obese boys were higher than in lean boys (Table 1 and Figure 1). Also serum fT_4_ concentrations were higher in overweight/obese boys compared to their lean peers, while no significant difference in serum fT_3_ concentrations was observed among boys (Table 1). In the overweight/obese group, boys exhibited higher concentrations of serum TSH, fT_4_, and fT_3_ compared to girls (Table 1). The serum fT_3_ concentration in boys with SH was higher than in euthyroid boys and higher than in their female peers exhibiting SH (Table 2).

Overweight/obese children and adolescents had higher WHtR (p<0.0001) compared with their lean peers. In the overweight/obese group, boys had a significantly higher BMI SDS than girls, while there was no sex difference in WHtR among the overweight/obese children. Children and adolescents having SH had higher BMI SDS (p<0.0001) and WHtR (p<0.0001) compared with the euthyroid study participants (Table 2). In a multivariate regression model including all 3006 lean and overweight/obese children and youths, serum TSH concentrations were associated positively with WHtR (p=0.0009) but not with BMI SDS (p=0.83), age (p=0.18), sex (p=0.23), or pubertal developmental stage (p=0.82). In the overweight/obese group, serum TSH concentrations were associated positively with BMI SDS and WHtR separately before and after adjustment for age, sex, and pubertal developmental stage in univariate regression models (Table 3). A positive correlation was observed in the overweight/obese group between serum fT_4_ concentrations and both BMI SDS and WHtR, but only the association between serum fT_4_ and WHtR remained significant after adjusting for age, sex, and pubertal developmental stage (Table 4). No associations between serum fT_3_ and anthropometrics were found among the overweight/obese study participants (Table 5).

When separately comparing BMI SDS and WHtR with the presence of SH in all the 3006 study participants in a multiple logistic regression model adjusted for age, sex, and pubertal development stage, the OR of exhibiting SH was 1.8 when being either overweight/obese compared to lean [95% confidence interval (CI): (1.3; 2.6), p=0.0007] or when presenting with a WHtR>0.5 compared to the study participants having a WHtR<0.5 [95% CI: (1.3; 2.6), p=0.0003].

## DISCUSSION

In this cross-sectional study on Danish children and adolescents, we observed a higher prevalence of SH among overweight/obese compared to lean children and adolescents. Concentrations of fasting serum TSH were associated positively with both BMI SDS and WHtR in the overweight/obese group. Independent of the degree of obesity, WHtR appeared to have a higher impact on the concentration of serum TSH compared with BMI SDS. Furthermore, serum fT_4_ concentrations correlated positively with WHtR but not with BMI SDS, while no significant associations were found between serum fT_3_ and either of the anthropometric measures. The 10.4% prevalence of SH in study participants with overweight/obesity is in accordance with reports from previous comparable studies, where prevalence rates of 7 to 23% have been shown (29). The lower prevalence of SH in the lean group was likewise comparable to results of other studies (7,9).

The higher concentrations of serum TSH observed among overweight/obese individuals compared with lean ones have similarly been observed in other studies conducted on overweight/obese children and adolescents (5,6,7,8,9,10,14). The literature is less univocal regarding data on the concentrations of peripheral thyroid hormones. Mostly, elevated concentrations of TSH have been reported in combination with elevated concentrations of fT_3_ (7,10,11,14) and slightly decreased (7) or normal fT_4_ concentrations (10,11,14). This is partly in contrast to our results where elevated serum fT_4_ concentrations were found among overweight/obese compared to lean children and adolescents, whereas serum fT_3_ concentrations did not differ between the two groups. fT_3_ is known to be the physiologically active peripheral thyroid hormone with approximately 80% of circulating T_3_ derived by extrathyroidal monodeiodination of T_4_ (30). A change in the monodeiodination pathway leading to augmented concentrations of active fT3, possibly resulting in an increased energy expenditure, has been suggested as an explanation of the thyroid hormone derangements in obesity (7,14,30). A disturbance in the negative feedback mechanism regulating the hypothalamus-pituitary-thyroid axis has been proposed as another explanation (30). With the observed elevated serum concentrations of TSH and fT_4_ among overweight/obese compared with lean children in the present study, our results support the latter notion, as people with a normal feedback mechanism and elevated concentrations of serum TSH would be expected to have decreased concentrations of peripheral thyroid hormones. The lacking consistency when evaluating peripheral thyroid hormones in obesity could be due to differences in study populations, as it has been proposed that thyroid function differs between lower grades of overweight and morbid obesity (31). The inconsistency could also point towards the elevated serum TSH concentrations to be the most pertinent thyroid disturbance in obesity. This possibility has previously been considered in a study of 226 obese and 39 lean adults, where serum TSH was associated with BMI and WC (32). TSH has been shown to induce adipogenesis and adipokine production (e.g. leptin) directly through TSH receptors in adipose tissue, independent of the mediating influence of thyroid hormones on energy balance (33). Likewise, signalling via leptin receptors has been shown to affect the TSH secretion by stimulating pro-thyrotropin-releasing hormone receptors in hypothalamus (34). The bidirectional signalling indicates a direct interaction between TSH and adipose tissue regulated differently from the hypothalamus-pituitary-thyroid axis and underlines the possibility that an elevated serum TSH concentration in obesity is more crucial than the varying derangements in peripheral thyroid hormone concentrations.

In line with the elevated serum concentrations of TSH in children with obesity, a positive association between TSH and BMI SDS has almost consistently been reported in previous pediatric studies (6,7,9,11,14,35). We observed a positive association between serum TSH and BMI SDS as well as between BMI SDS and serum fT_4_, whereas serum fT_3_ did not correlate with BMI SDS. Previously, a positive association between BMI SDS and serum total T_4_ has been reported in 190 obese children as compared to 133 age- and sex-matched controls, while no significant associations between BMI SDS and serum fT_4_ or serum fT_3_ (9) were observed, the latter being compatible with our results.

Few studies have focused on the correlation between thyroid variables and anthropometrics other than BMI. A positive association between serum TSH and WC has been reported in 201 adult euthyroid women (16), whereas another study failed to show any association in 703 multiethnic obese children and adolescents (3). In a study of 240 pre-pubertal children, serum fT_3_ concentrations were strongly influenced by augmented central fat accumulation, measured by dual-energy x-ray absorptiometry (DXA), independent of total body fat mass and percentage of body fat. Augmented visceral fat accumulation is associated with the development of several cardio-metabolic risk factors (15,18). BMI SDS is of limited use in the evaluation of body composition due to its inability to differentiate muscle mass from bone and fat mass (36). WHtR takes body composition into account by evaluating WC in accordance to variability in height, and therefore WHtR is considered to be a more appropriate proxy of central adiposity compared with BMI SDS (15,18,19). Conceivably, in the present study, we included WHtR as a surrogate measure of central adiposity. We observed that serum TSH associated positively with WHtR and BMI SDS among overweight/obese individuals, but when all study participants were included in the analysis, serum TSH only showed a significant association with WHtR. Additionally, the stronger determinant of serum fT_4_ concentrations appeared to be the WHtR among the overweight/obese children and adolescents. This finding suggests that an augmented WHtR, and thus central obesity, is associated with the risk of thyroid dysfunctions.

A sex difference was demonstrated in the overweight/obese group, as overweight/obese boys showed higher concentrations of thyroid hormones compared with overweight/obese girls. Even though we did not see a sex-specific difference in WHtR, it is possible that the higher concentrations of thyroid hormones in boys can be explained by a correlation between thyroid hormones and the degree of central obesity, as it has been suggested that men accumulate more visceral fat than women, independent of total body fat mass (37). The proposed difference between men and women regarding accumulation of visceral fat is biologically plausible for boys and girls as well. We also observed serum TSH concentrations to be higher in obese compared to overweight and lean girls, while serum TSH concentrations in obese and overweight boys were higher than in lean boys. This indicates that girls need to become relatively more overweight than boys before they show corresponding elevated concentrations of TSH. The theory of visceral fat to accumulate faster among boys compared to girls (37) could contribute to this observed sex difference in thyroid-pituitary gland function. Nevertheless, the mechanisms explaining thyroid abnormalities in obesity are still unclear. Due to the cross-sectional nature of this study, we are unable to clarify whether the higher prevalence of SH among children and adolescents with overweight/obesity plays a pathogenic part in obesity or if rather it reflects an adaptive effort to raise energy expenditure.

One limitation of this study is the lack of thyroid antibody measurements and the possibility that children with autoimmune thyroiditis may have been included in the study sample. However, this is not very likely, since all patients in the overweight/obese group were evaluated clinically by a trained pediatrician regarding their clinical thyroid status. Furthermore, other studies conducted on obese children and adolescents have reported positive results for thyroid antibodies in 5.7% to 19.5% of obese children with SH, concluding that autoimmune thyroiditis is rarely the cause of elevated thyroid hormone concentrations in obesity (4,5,9). The relative limitation of blood samples to be analyzed for serum fT_3_ concentrations is another shortcoming of the present study. Also, the change in method used to analyze fT_4_ and TSH concentrations is a limitation. We relied on the quality control performed by our laboratory at the time of method change which determined reliable correlation coefficients and comparable coefficients of variations between the analyzers. Therefore, we believe that the fT_4_ and TSH concentrations measured by the two different analyzers are comparable. The maximum interval of 60 days between the obtaining of anthropometric measurements and the blood sampling allowed for the variability caused by the possibility that overweight/obese children enrolled in obesity treatment lost or gained weight before the blood sample was taken. This could mean that the presented result is a conservative estimate of the SH prevalence among the overweight/obese group. Yet, an additional limitation of the study is the self-reported staging of pubertal development of the controls. Tanner staging is by its nature an arbitrary measure as it attempts to characterize a continuous developmental progress into one of five distinct stages and therefore both objectively measured and self-reported staging needs to be carefully interpreted. The primary strength of this study is that it was conducted on a relatively large study population consisting of well-characterized overweight/obese and lean children and adolescents.

In conclusion, this study shows that SH is more prevalent among children and adolescents with obesity compared to their lean peers. Central fat accumulation is related to the thyroid abnormality independent of the degree of overall obesity, indicating central obesity as an aggravated risk for development of SH. Nevertheless, the causality of the association remains to be clarified and longitudinal studies are needed to understand possible links between SH and central fat accumulation.

## Figures and Tables

**Table 1:**
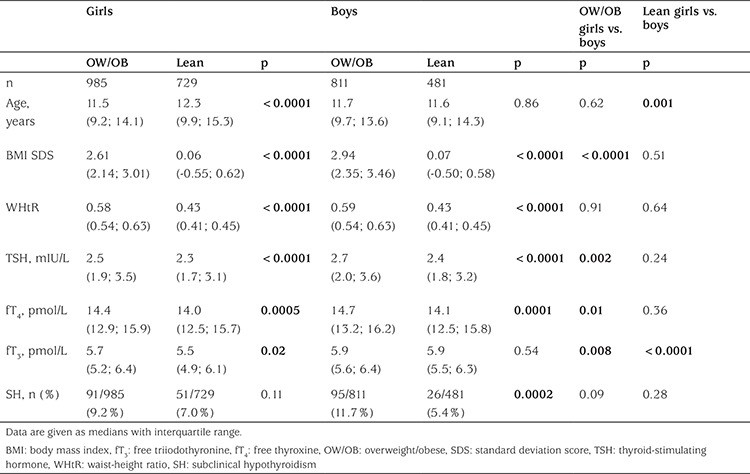
Baseline characteristics of 1210 lean and 1796 overweight/obese Danish children and adolescents

**Table 2:**
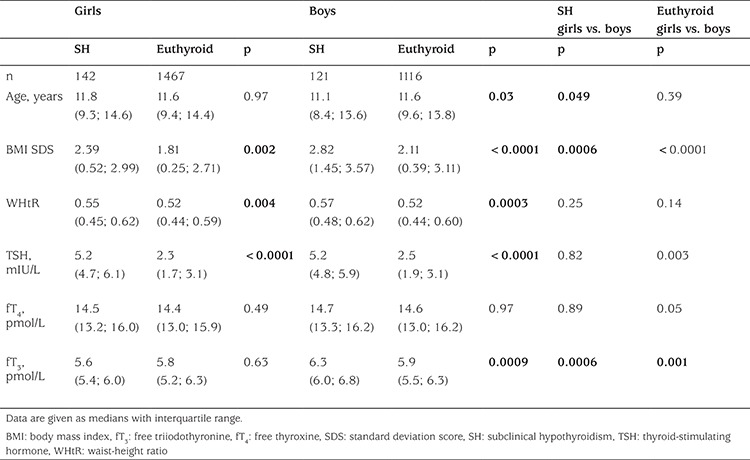
Baseline characteristics of Danish girls and boys exhibiting subclinical hypothyroidism compared with euthyroid girls and boys

**Table 3:**
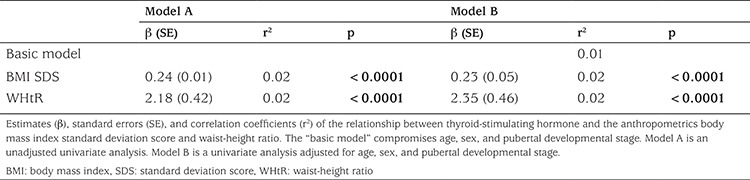
Multiple linear regressions showing the relationship between fasting serum thyroid-stimulating hormone concentrations and anthropometrics (n=1796) in the overweight/obese group

**Table 4:**
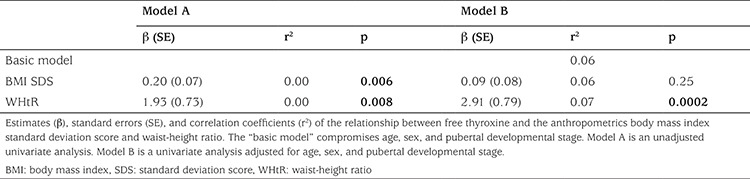
Multiple linear regressions showing the relationship between fasting serum free thyroxine concentrations and anthropometrics (n=1796) in the overweight/obese group

**Table 5:**
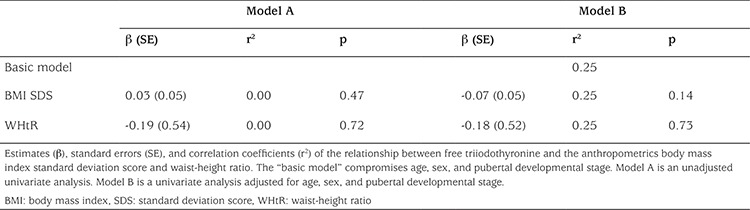
Multiple linear regressions showing the relationship between fasting serum free triiodothyronine concentrations and anthropometrics (n=440) in the overweight/obese group
